# Supplementary material for: Exploring the Drought Tolerant Quantitative Trait Loci in Spring Wheat
Source: Plants (Basel). 2024 Mar 21;13(6):898. doi: 10.3390/plants13060898 (PMC10975456; doi:10.3390/plants13060898)
Supplement: Supplementary file 1 [file plants-13-00898-s001.zip › Supplementary figure.docx]

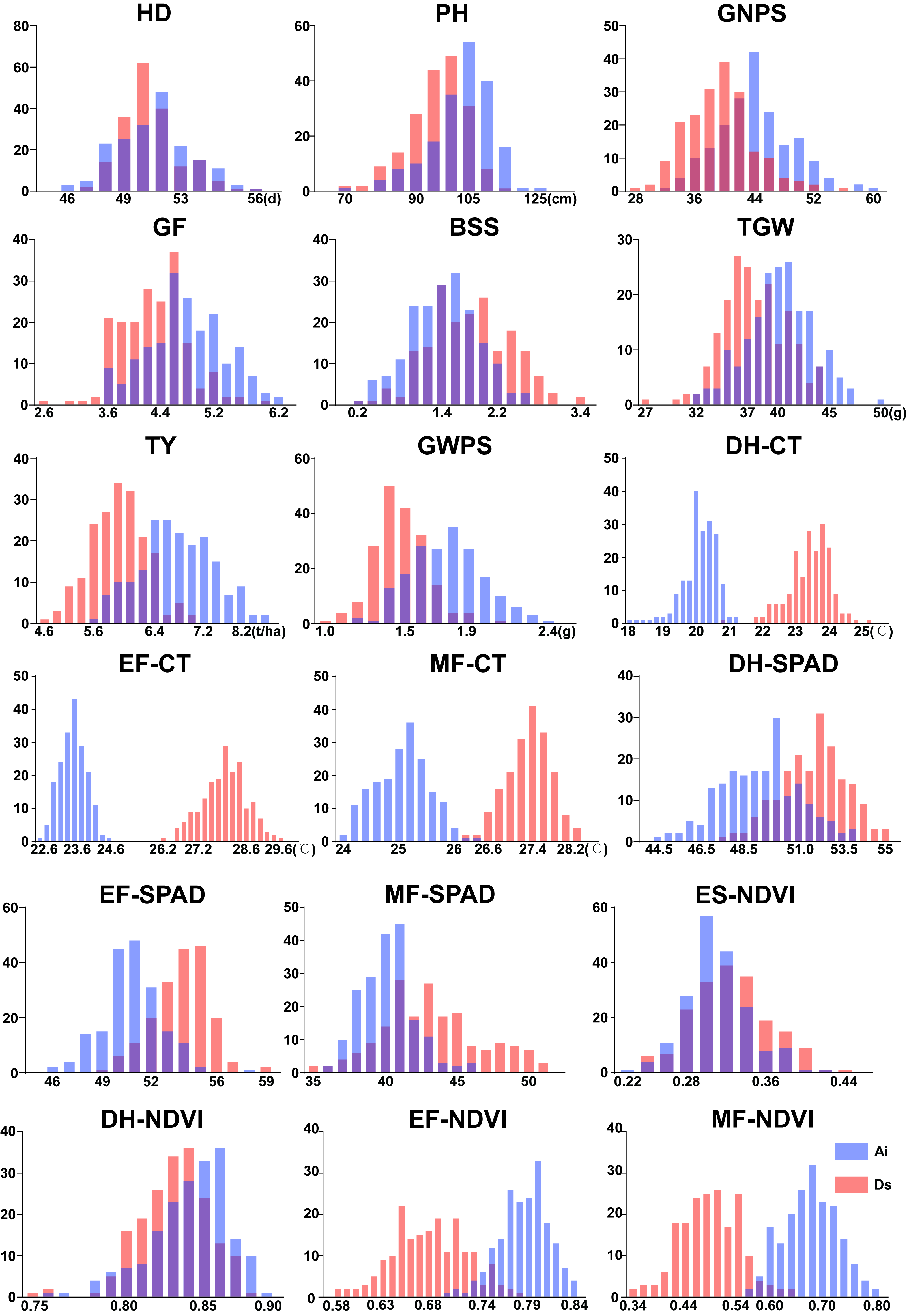


**Figure S1 The Frequency distribution of 186 RILs in different treatments.** HD, heading date; PH, plant height; GNPS, grain number per spike; GF, grain fullness; BSS, number of basal sterile spikelet; TGW, thousand grain weight; GWPS, grain weight per spike; TY, total yield; CT, canopy temperature; SPAD, chlorophyll content; NDVI, normalized differential vegetation index; EF, early filling stage; MF, middle filling stage; ES, elongation stage. Ai, Adequate irrigation; Ds, Drought stress.
