# Supplementary figures and images for: Exploring the Drought Tolerant Quantitative Trait Loci in Spring Wheat
Source: Plants (Basel). 2024 Mar 21;13(6):898. doi: 10.3390/plants13060898 (PMC10975456; doi:10.3390/plants13060898)

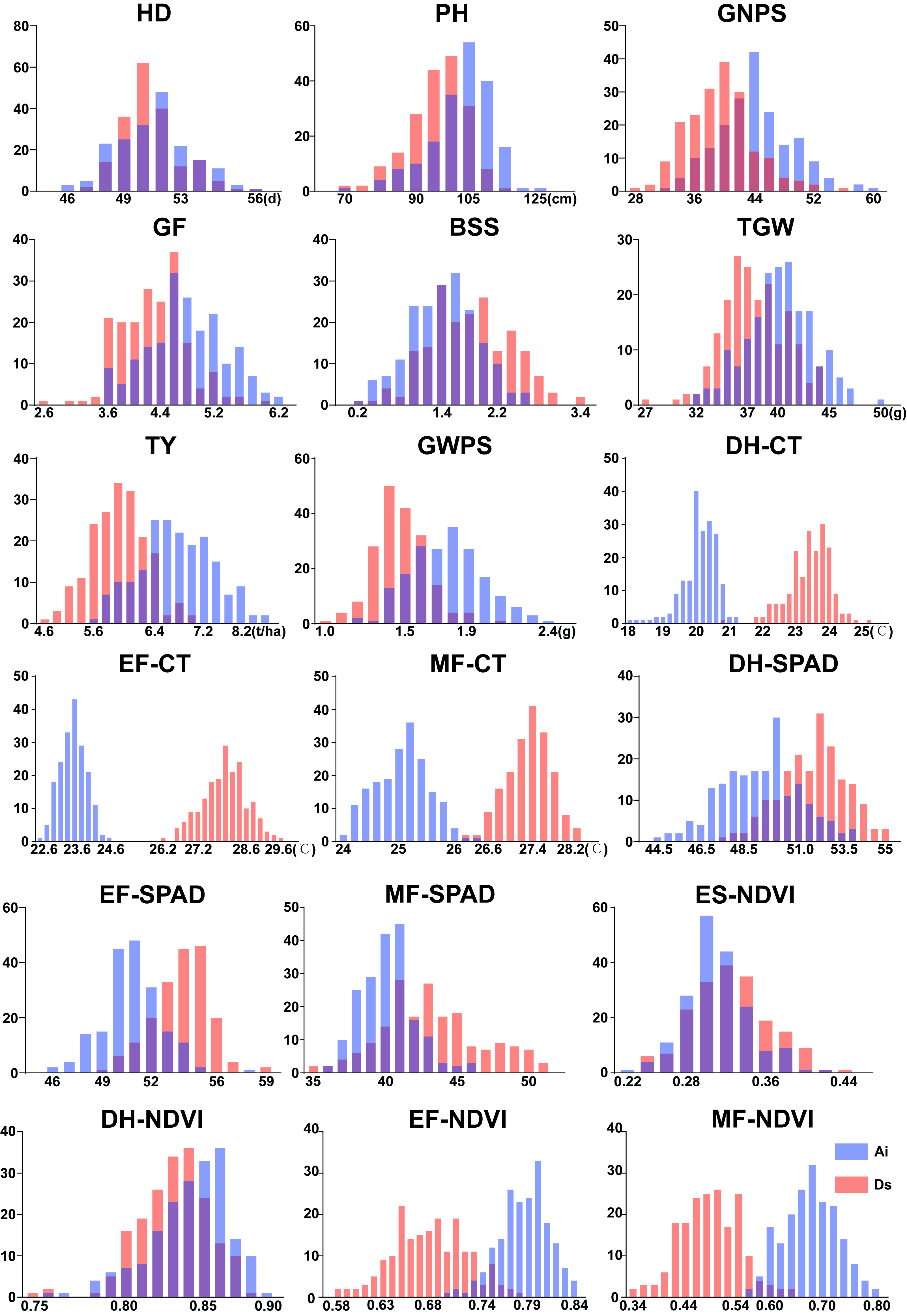

Supplement: Supplementary file 1 [file plants-13-00898-s001.zip › Supplementary figure.tif]
